# Evaluation of putative reference genes for gene expression normalization in soybean by quantitative real-time RT-PCR

**DOI:** 10.1186/1471-2199-10-93

**Published:** 2009-09-28

**Authors:** Ruibo Hu, Chengming Fan, Hongyu Li, Qingzhu Zhang, Yong-Fu Fu

**Affiliations:** 1 Institute of Crop Science, National Key Facility of Crop Gene Resource and Genetic Improvement, Chinese Academy of Agricultural Sciences, Beijing 100081, PR China

## Abstract

**Background:**

Real-time quantitative reverse transcription PCR (RT-qPCR) data needs to be normalized for its proper interpretation. Housekeeping genes are routinely employed for this purpose, but their expression level cannot be assumed to remain constant under all possible experimental conditions. Thus, a systematic validation of reference genes is required to ensure proper normalization. For soybean, only a small number of validated reference genes are available to date.

**Results:**

A systematic comparison of 14 potential reference genes for soybean is presented. These included seven commonly used (*ACT2, ACT11, TUB4, TUA5, CYP, UBQ10, EF1b*) and seven new candidates (*SKIP16, MTP, PEPKR1, HDC, TIP41, UKN1, UKN2*). Expression stability was examined by RT-qPCR across 116 biological samples, representing tissues at various developmental stages, varied photoperiodic treatments, and a range of soybean cultivars. Expression of all 14 genes was variable to some extent, but that of *SKIP16, UKN1 *and *UKN2 *was overall the most stable. A combination of *ACT11, UKN1 *and *UKN2 *would be appropriate as a reference panel for normalizing gene expression data among different tissues, whereas the combination SKIP16, UKN1 and MTP was most suitable for developmental stages. *ACT11, TUA5 *and *TIP41 *were the most stably expressed when the photoperiod was altered, and *TIP41, UKN1 *and *UKN2 *when the light quality was changed. For six different cultivars in long day (LD) and short day (SD), their expression stability did not vary significantly with *ACT11, UKN2 *and *TUB4 *being the most stable genes. The relative gene expression level of *GmFTL3*, an ortholog of Arabidopsis *FT *(*FLOWERING LOCUS T*) was detected to validate the reference genes selected in this study.

**Conclusion:**

None of the candidate reference genes was uniformly expressed across all experimental conditions, and the most suitable reference genes are conditional-, tissue-specific-, developmental-, and cultivar-dependent. Most of the new reference genes performed better than the conventional housekeeping genes. These results should guide the selection of reference genes for gene expression studies in soybean.

## Background

Gene expression analysis plays an important role in furthering our understanding of the signalling and metabolic pathways which underlie developmental and cellular processes. Real-time quantitative reverse transcription PCR (RT-qPCR) represents a particularly suitable technology platform for this purpose, thanks to its sensitivity, specificity, dynamic range and high throughput capacity [1-4]. To avoid experimental errors arising from variation in the quantity and integrity of the RNA template, as well as in the efficiency of the RT reaction used to synthesize cDNA, a normalization step is an essential pre-requisite. The most common way to achieve normalization is to include one, or a small number of reference genes, whose expression is assumed to be constitutive [5-7]. Such genes are expressed at a constant level in all tissues independent of the growing environment [1,5-8]. Commonly used reference genes include ribosomal RNA (*18SrRNA*) and a number of housekeeping genes, such as those encoding actin (*ACT*), tubulin (*TUB*), glyceraldehyde-3-phosphate dehydrogenase (*GAPDH*), polyubiquitin (*UBQ*) and elongation factor 1-α (*EF1α*) [1,6,9,10]. Typically, these genes have been simply assumed to be constitutively expressed, as they are involved in basic and ubiquitous cellular processes [1,5,9,11]. However, the evidence is that transcript levels of housekeeping genes can vary considerably in response to changes in experimental conditions and/or tissue types, so that none of the commonly exploited genes can be viewed as a universal reference. Instead, the onus is on the experimenter to select a panel of genes which is appropriate for the specific set of chosen experimental conditions and tissue types [7,8,12-14]. In many cases, a single reference gene is inadequate, and any such reliance is likely to produce erroneous conclusions vis-à-vis expression patterns [15-18].

The importance of expression stability in the choice of reference genes is high enough to have prompted the development of software packages, such as geNorm [19] and NormFinder [20], to identify them [17,21]. A number of reference gene validation attempts have been reported [22-29], and in plants specifically, these have covered both model and crop species: *Arabidopsis thaliana *[9,30], rice [31,32], *Brachypodium sp*. [33], wheat [34], barley [35], soybean [36,37], tomato [38], potato [39], sugarcane [40], grape [16] and poplar [15,41]. The *A. thaliana *ATH1 array has been used to identify a set of reference genes superior to the conventionally applied housekeeping genes [9], and the wider relevance of this set has been demonstrated in *Brachypodium sp*. [33], tomato [38], grape [16] and poplar [15].

Soybean is the leading legume crop, and has been used as a model plant in the context of the flowering response to photoperiod. Many of these studies have used *TUB *and/or *ACT *as a reference gene (Additional file 1). A literature search based on the keywords "soybean" and "gene expression" produced 54 hits in PubMed (publication period 2001 to 2009). In 23 of these studies (43%), *TUB *was the reference gene, in 15 of them (28%) *ACT*, and in six (11%) *18SrRNA*. All of the studies surveyed used one single reference gene and no preliminary validations were performed (Additional file 1). To date, only a limited number of statistically validated reference genes have been identified in soybean. A comparison of the performance of ten conventional housekeeping genes across 21 soybean samples allowed the identification of a panel of genes suitable for gene expression normalization [36]. However, the limited number of samples tested meant that a full representation of developmental stages and tissues/organs could not be achieved; instead, a set of new reference genes, chosen to exhibit constancy of expression over a range of experimental conditions, was mined from multiple soybean microarray datasets [37]. In the present report, we compare the performance of seven commonly used housekeeping genes and seven of these new reference genes across a large set of biological samples representing various developmental stages, tissues, photoperiod treatments and cultivars of soybean. The recently released soybean whole genome sequence [42] has facilitated genome-wide mining for reference genes in soybean. Based on sequence homology, soybean orthologs of the best three A. thaliana reference genes have been identified. A further four genes have been selected, which have shown stable expression on a micro-array platform [37]. Our data indicate that many of these newer reference genes indeed have greater expression stability than the conventionally used housekeeping genes. As a result, the use of combinations of these reference genes should provide a more reliable means of normalizing gene expression.

## Results

### Transcription profiling of soybean reference genes

**Figure 1:**
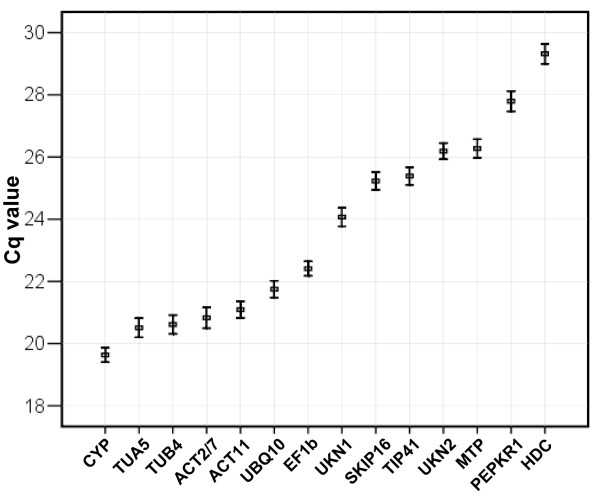
**Expression levels of the candidate reference genes across experimental samples**. Values are given in the form of RT-qPCR quantification cycle numbers (Cq values). The boxes represent mean Cq values, the bars standard deviations.

A RT-qPCR assay based on SYBR Green detection was carried out to examine the stability of the expression of the 14 candidate genes (Table 1). The full sample set was included in each technical replicate to exclude any artefacts due to between-run variation. Each RT reaction was repeated once, and three independent technical replicates were performed for each experiment. The expression level of the candidate reference genes are presented as quantification cycle (Cq) values (Figure 1). The mean Cq values of the genes ranged from 17 to 32, with most lying between 20 and 25. *CYP *was the most highly expressed of the set, with a mean Cq of 19.6, and *HDC *the least (mean Cq of 32.7). *EF1b *showed the least variation (CV of 5.6%), while *ACT2/7 *(7.3%) and *TUB4 *(7.7%) were the most variable. The variation in Cq is illustrated as a scatter diagram in Additional file 2.

**Table 1:** Reference genes used for gene expression normalization in soybean.

**Gene symbol**	**Gene locus**	**NCBI Accession No**.	**Unigene ID**	**Arabidopsis ortholog locus**	**Arabidopsis locus description**	**Function**
*ACT11*	Glyma18g52780.1	BW652479	Gma.32186	AT3G12110	Actin 11	Cytoskeletal structural protein
*ACT2/7*	Glyma04g39380.1	BW677100	Gma.30938	AT5G09810	Actin 2/7	Cytoskeletal structural protein
*CYP*	Glyma12g02790.1	CF806591	Gma.31618	AT2G21130	Cyclophilin	Protein folding
*EF1b*	Glyma02g44460.1	EV279336	Gma.2137	AT5G12110	Elongation factor 1β	Translational elongation
*TUA5*	Glyma05g29000.1	CA801144	Gma.13580	AT5G19780	alpha Tubulin	Structural constituent of cytoskeleton
*TUB4*	Glyma03g27970.1	EV263740	Gma.31016	AT5G12250	beta Tubulin	Structural constituent of cytoskeleton
*UBQ10*	Glyma07g32020.1	EH258122	Gma.17451	AT4G05320	Ubiquitin 10	Protein binding, protein modification
*HDC*	Glyma08g05480.1	CK768960	Gma.34482	AT1G58050	Nuclear helicase	Unwinding of the DNA double-helix
*SKIP16*	Glyma12g05510.1	CD397253	Gma.6079	AT1G06110	SKP1/Ask-Interacting Protein 16	Protein binding
*MTP*	Glyma03g29350.2	CF808703	Gma.7635	AT2G41790	Metalloprotease, Insulin degrading enzyme	Protein degradation
*PEPKR1*	Glyma10g38460.1	AW396185	Gma.23799	AT1G12580	Phosphoenolpyruvate Carboxylase-Related Kinase 1	Protein phosphorylation
*TIP41*	Glyma20g26690.1	EV263725	Gma.10647	At4G34270	TIP41-like family protein	TOR (Target of Rapamycin) signalling element
*UKN1*	Glyma12g02310.1	BU578186	Gma.32694	AT3G13410	Hypothetical protein	Unkown
*UKN2*	Glyma06g04180.1	BE330043	Gma.20882	AT4G33380	Hypothetical protein	Unkown

**Figure 2:**
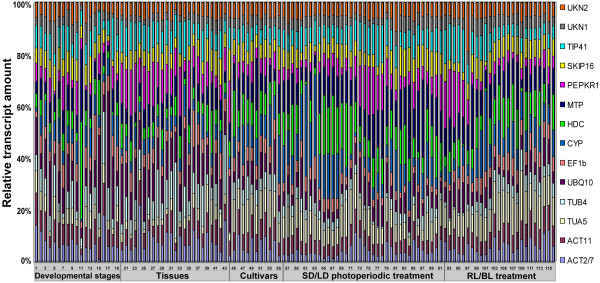
**Distribution of relative transcript quantities of the reference genes across all samples**. Transcript quantities are represented as percentages of the aggregated 14-transcript pool for each sample. 1-20: across various developmental stages; 21-44: across different tissues; 45-56: across cultivars; 57-92: response to short day (SD) and long day (LD) photoperiods; 93-116: response to exposure to red (RL) and blue (BL) light. Detailed sample information given in Additional file 5.

The variation in relative transcript quantity of the reference genes across all samples is shown as Figure 2. Here, transcript quantities are represented as percentages, relative to the aggregated reference transcript pool of each sample. The proportion of *SKIP16, UKN2 *and *UKN1 *transcript remained relatively constant across samples, while those of *HDC *and *TUB4 *were rather variable, especially with respect to developmental stage and tissue type. Although the expression level of *UKN2 *was fairly constant among almost all the samples, its expression was particularly low in the 2^nd ^triofoliolate at the stage when the 3^rd ^triofoliolate fully expanded. In contrast, the expression of *HDC *was particularly high in this tissue/developmental stage combination. *TUA5 *expression varied widely across developmental stages and tissue types, but was largely unaffected by photoperiodic treatment or cultivar. Thus, the transcript level of none of the reference genes was truly constant, rather it varied both temporally and spatially.

### PCR efficiency analyses

Melting curve analyses were performed following the RT-qPCR. The specificity of the amplicons was confirmed by the presence of a single peak (a representative trace is shown as Additional file 3). Electrophoretic separation of the amplicons produced a single fragment of the expected size in all cases, with no visible primer-dimer products. Five primer pairs were designed either to span an intron, or to target exon-exon junctions (Table 2), and used to compare amplicons derived from genomic DNA template with those from cDNA template. This comparison demonstrated that the cDNA template was free of contaminating gDNA. No amplification was detectable in the absence of template. Standard curves were generated using a ten-fold serial dilution of a cDNA pool, and these enjoyed a linear correlation coefficient (R2) of 0.994-0.999. Based on the slopes of these standard curves, the estimated PCR amplification efficiencies ranged from 94% to 106% (Table 2 and Additional file 4).

**Table 2:** Reference gene primer sequences and amplicon characteristics.

**Gene symbol**	**Forward primer sequence [5'-3']**	**Reverse primer sequence [5'-3']**	**Positions in cDNA**	**Amplicon length (bp)**	**Tm (°)**	**PCR efficiency (%)**	**Regression coefficient (R^2^)**
*ACT11*	ATCTTGACTGAGCGTGGTTATTCC	GCTGGTCCTGGCTGTCTCC	Exon3/Exon3	126	83.3	104	0.998
*ACT2/7*	AATTCACGAGACCACCTACAAC	TGAGCCACCACTAAGAACAATG	Exon3/Exon3	91	78.8	98	0.999
*CYP*	ACGACGAAGACGGAGTGG	CGACGACGACAGGCTTGG	Exon	130	87.8	96	0.999
*EF1b*	CCACTGCTGAAGAAGATGATGATG	AAGGACAGAAGACTTGCCACTC	Exon4/Exon5	134	82.0	94	0.998
*TUA5*	TGCCACCATCAAGACTAAGAGG	ACCACCAGGAACAACAGAAGG	Exon6/Exon7	103	81.0	104	0.999
*TUB4*	GGCGTCCACATTCATTGGA	CCGGTGTACCAATGCAAGAA	Exon2/Exon2	111	83.8	106	0.999
*UBQ10*	TCCCACCAGACCAGCAGAG	CACGAAGACGCAACACAAGG	Exon	117	84.0	98	0.999
*HDC*	AGGTCGTTGTTGTCTCAGGTG	CGTGCCGCTTCAGTCTCAG	Exon6/Exon6	88	80.0	95	0.999
*SKIP16*	GAGCCCAAGACATTGCGAGAG	CGGAAGCGGAAGAACTGAACC	Exon1/Exon1	60	80.8	102	0.999
*MTP*	CGCTCCAAGTGCTCCTCATTAG	TGAAGTAACCGACGCCAACG	Exon1/Exon2	71	82.8	93	0.999
*PEPKR1*	AGCAACCAAACAAATCCTGAACAAC	CCAACATCCAACTCTCCACAACC	Exon6/Exon6	68	75.6	98	0.995
*TIP41*	AGGATGAACTCGCTGATAATGG	CAGAAACGCAACAGAAGAAACC	Exon5/Exon6	88	77.8	105	0.997
*UKN1*	TGGTGCTGCCGCTATTTACTG	GGTGGAAGGAACTGCTAACAATC	Exon1/Exon1	74	78.3	96	0.994
*UKN2*	GCCTCTGGATACCTGCTCAAG	ACCTCCTCCTCAAACTCCTCTG	Exon5/Exon6	79	79.5	93	0.999

### Gene expression stability analyses

**Figure 3:**
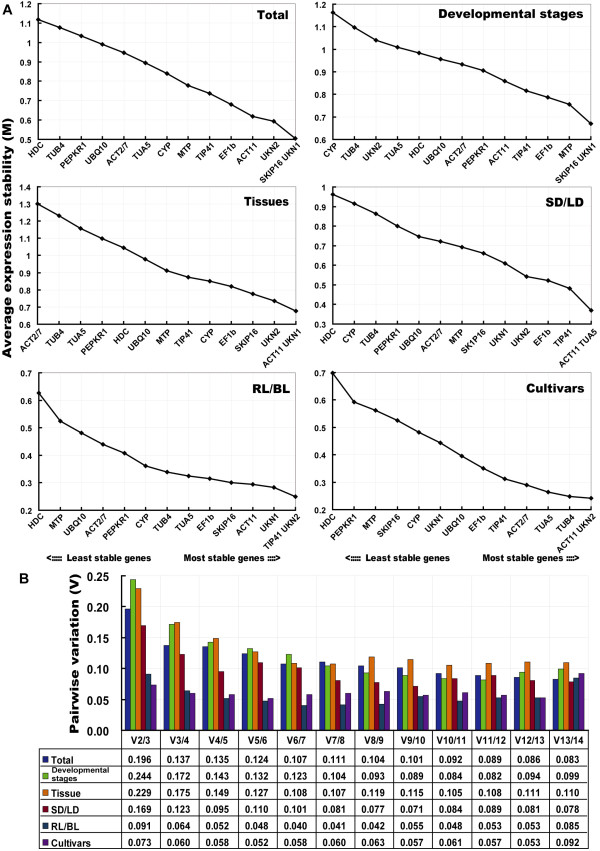
**Gene expression stability and pairwise variation of the candidate genes as predicted by geNorm**. A. Mean expression stability (M) following stepwise exclusion of the least stable gene across all treatment groups. The least stable genes are on the left, and the most stable on the right. B. The optimal number of reference genes required for effective normalization. The pairwise variation (Vn/Vn+1) was analyzed between the normalization factors NFn and NFn+1 by geNorm program to determine the optimal number of reference genes required for RT-qPCR data normalization.

The expression stability of the set of candidate reference genes was examined by geNorm software, which calculates, for each gene, a measure of its expression stability (M) based on the average pairwise variation between all genes tested (Figure 3). Stepwise exclusion of the least stable gene allowed the genes to be ranked according to their M value (the lower the M value, the higher the gene's expression stability) [17], as depicted in Figure 3A. All the genes had an M value below the geNorm threshold of 1.5. Across all the samples, *SKIP16 *and *UKN1 *were the most stably expressed, and *HDC *the least. As a result, the latter was the first to be excluded from the analysis (Figure 3A). Among the various developmental stages, *SKIP16 *and *UKN1 *remained the most stable, and *CYP *the least stable. *ACT11 *and *UKN1 *were the most highly ranked across the set of tissues at the various developmental stages, while *ACT2/7 *was the least stable. In response to the short day (SD) and long day (LD) treatments, *ACT11 *and *TUA5 *were the most stable genes, and *HDC *the least; while in response to blue light (BL) and red light (RL) treatment, *TIP41 *and *UKN2 *were the most stable, and *HDC *the least.

To determine the optimal number of genes required for normalization, geNorm was used to calculate the pairwise variation (Vn/Vn+1) between sequential normalization factors (NF) (NFn and NFn+1) [17]. As reported by Vandesompele et al (2002), a threshold value of 0.15 was adopted [17]. In the SD/LD comparison, three genes was sufficient for normalization, since the V3/4 value was <<0.15 (Figure 3B). Differences in the expression stability of the candidate reference genes were less marked in the RL and BL photoperiodic treatment series, than in the other series (Figure 3). The V2/3 value for the RL/BL comparison was 0.091, so that *TIP41 *together with *UKN2 *would be sufficient for normalization purposes. Among the cultivars, the pair *ACT11 *and *UKN2 *produced a V2/3 value of 0.073. However, for the comparisons based on developmental stage and tissue type, four genes were necessary, since the V3/4 values lay above the threshold. When all the experimental samples were considered together, the V2/3 value was 0.196 and the V3/4 was 0.137, suggesting that the addition of a fourth gene did not improve the quality of the normalization (Figure 3B). Overall, the combination *SKIP16, UKN1 *and *UKN2 *was appropriate for all sets of samples.

Stability of expression was then re-analysed using the program NormFinder, which is based on a variance estimation approach [21], and ranks the genes according to their stability under a given set of experimental conditions. The ranking generated by this approach was slightly different from that determined by geNorm (Table 3). *ACT11 *and *UKN1 *were still ranked the highest for tissue samples, and *ACT11 *and *UKN2 *the highest for inter-cultivar comparisons. *HDC, CYP *and *ACT2/7 *ranked consistently poorly. Among developmental stages, *EF1b *and *MTP *emerged as the most stably expressed (ranked second and third by geNorm) (Figure 3). *ACT11 *and *TUA5 *were identified by both NormFinder and geNorm as being among the three most stable genes under SD and LD treatments. When evaluated across all the experimental samples, the same four genes were identified by both programs, although their rank order was slightly altered.

**Table 3:** Expression stability of the reference genes, as calculated by NormFinder.

**Rank**	**Total**		**Developmental stage**		**Tissues**		**SD/LD**		**RL/BL**		**Cultivars**	
	**Gene**	**Stability**	**Gene**	**Stability**	**Gene**	**Stability**	**Gene**	**Stability**	**Gene**	**Stability**	**Gene**	**Stability**
1	*UKN2*	0.3513	*EF1b*	0.4996	*ACT11*	0.4571	*ACT11*	0.2849	*ACT11*	0.1441	*UKN2*	0.1632
2	*ACT11*	0.3716	*MTP*	0.5625	*UKN2*	0.5193	*UKN2*	0.3787	*SKIP16*	0.1845	*ACT11*	0.2092
3	*UKN1*	0.4685	*TIP41*	0.5632	*UKN1*	0.5881	*TUA5*	0.4561	*UKN1*	0.1911	*TUB4*	0.2188
4	*SKIP16*	0.5131	*ACT11*	0.6258	*SKIP16*	0.6302	*UKN1*	0.4743	*EF1b*	0.2058	*TUA5*	0.3022
5	*EF1b*	0.6069	*UKN1*	0.6261	*EF1b*	0.6360	*EF1b*	0.5074	*TIP41*	0.2217	*UKN1*	0.3443
6	*TIP41*	0.6300	*SKIP16*	0.7082	*TIP41*	0.6667	*TIP41*	0.5248	*UKN2*	0.2408	*TIP41*	0.3500
7	*MTP*	0.7137	*UBQ10*	0.7902	*MTP*	0.7518	*SKIP16*	0.5437	*TUA5*	0.3041	*ACT2/7*	0.3548
8	*ACT2/7*	0.9339	*TUA5*	0.8033	*CYP*	0.8712	*MTP*	0.5837	*TUB4*	0.3609	*EF1b*	0.4823
9	*CYP*	0.9449	*ACT2/7*	0.8627	*TUA5*	1.0785	*ACT2/7*	0.6481	*CYP*	0.4132	*SKIP16*	0.4901
10	*UBQ10*	0.9540	*HDC*	0.8831	*UBQ10*	1.1184	*UBQ10*	0.7267	*PEPKR1*	0.4929	*MTP*	0.5359
11	*TUA5*	0.9737	*PEPKR1*	0.8925	*HDC*	1.2256	*TUB4*	0.9931	*ACT2/7*	0.5374	*PEPKR1*	0.5946
12	*PEPKR1*	1.0761	*UKN2*	0.9872	*PEPKR1*	1.2737	*PEPKR1*	1.0296	*MTP*	0.6254	*CYP*	0.6860
13	*TUB4*	1.1017	*TUB4*	1.1854	*TUB4*	1.3359	*CYP*	1.0575	*UBQ10*	0.6947	*UBQ10*	0.6909
14	*HDC*	1.1398	*CYP*	1.3691	*ACT2/7*	1.5145	*HDC*	1.0728	*HDC*	1.1874	*HDC*	1.2811

### Reference gene validation

**Figure 4:**
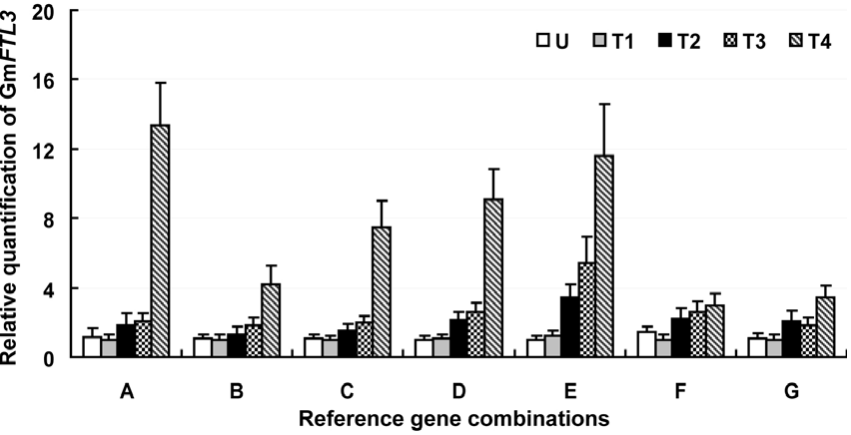
**Relative quantification of *GmFTL3 *expression using validated reference genes for normalization**. A: *SKIP16*; B: *UKN1*; C: *SKIP16 *and *UKN1*; D: *SKIP16*, *UKN1 *and *MTP*; E: *SKIP16*, *UKN1*, *MTP *and *EF1b*; F: *CYP*; G: *TUB4*. The results are represented as a mean fold change in relative expression compared to the first sampling stage (U). cDNA samples taken from the same set used for gene expression stability analysis: U, T1, T2, T3 and T4 indicate, respectively, the aerial part of plants collected at the full expansion of the unifoliolate, the first trifoliolate, the second trifoliolate, the third trifoliolate and the fourth trifoliolate leaf.

The expression pattern of *GmFTL3*, a soybean *FLOWERING LOCUS T *(*FT*) ortholog, was analysed using the selected reference genes (Figure 4). In *A. thaliana*, *FT *acts as a floral promoter and an integrator of various flowering pathways [43-47]. *GmFTL3 *has been proposed as a flowering promoter, since its ectopic over-expression in *A. thaliana *is associated with an extremely early flowering phenotype (unpublished data). Its pattern of expression was assessed at five distinct vegetative growth stages. When normalized using *SKIP16, UKN1, MTP *and *EF1b *as reference genes, transcript abundance gradually increased over time, peaking at the onset of flowering (the fourth trifoliolate leaf fully expanded) (Figure 4E). Similar expression patterns were generated when either three or two of the most stable genes (as identified by geNorm) were used for normalization (Figure 4C and 4D). When only one reference gene was employed, its expression was also rather similar to the above patterns (Figure 4A and 4B), but differences were evident in estimated transcript abundance, which was higher when normalized against *SKIP16 *than against *UKN1*, presumably because *UKN1 *transcript level was greater than that of *SKIP16 *(Figure 1). Normalization based on either of the less stable genes *CYP *or *TUB4 *produced a picture of GmFTL3 expression in which transcript level was constant during the vegetative growth stages (Figure 4F and 4G). Its relatively less abundant expression at the onset of flowering was a consequence of *CYP *and *TUB4 *up-regulation during this period. It suggested that not only the stability but also the abundance of a reference gene affected the normalized results.

## Discussion

Reference genes are routinely used as a means of quantifying gene expression. The ideal reference genes should be expressed at a constant level throughout the plant and not be influenced by exogenous treatment [1,5]. Housekeeping genes, such as those involved in basic cellular processes (*EF1α, UBQ *and *CYP*) or cell structure maintenance (*ACT, TUB*), have been extensively used, but increasingly it has become apparent that their expression level is not as independent of experimental conditions as had been expected [6-8,13,14,18,48]. This implies a need to test in advance the expression stability of any proposed reference gene(s), a procedure which is often not followed in the literature. Normalization based on several reference genes has begun to become the standard, supported by the development of software such as geNorm and Normfinder [17,21]. However, the prior validation of reference genes remains uncommon in plant research, although it is the norm in human and animal research [22-25,32,49-54].

Soybean has been used as a model plant for the study of photoperiod-induced floral induction [45], but the molecular mechanism underlying this induction remains poorly understood. In soybean, *ACT, TUB *and *UBQ *are the most frequently used reference genes (Additional file 1), but there is increasing evidence that their expression is not particularly stable under certain conditions. More recently, some alternative reference genes have emerged [36,37]. Although four of these (*SKIP16, MTP, PEPKR1 *and *UKN2*) have been shown by RT-qPCR to be stably expressed under certain limited experimental conditions, no detailed validation has to date been carried out to test their suitability in experiments involving photoperiodic treatments.

In the present study, we used more subdivided samples to make the data more representative (Additional file 5). To our knowledge, this is the first systematic study of the expression stability of reference genes across such a large number of samples under varied light regimes (SD/LD/DD/LL, RL and BL) in soybean. The 14 reference genes in general out-performed the conventional housekeeping genes, and the poor performance of commonly used genes such as *ACT2/7 *and *TUB4 *was of particular note (Figure 3). *SKIP16, UKN1 *and *UKN2 *were overall the most stable and were good candidates for the normalization of general gene expression. But different sets of samples had their own best reference genes (Figure 3). For example, *ACT11 *is one of best reference genes for both different tissue and photoperiod samples, whereas *TIP41 *did better than *ACT11 *when studying samples harvesting from different quality light (blue and red light) and *SKIP16 *was the best reference for developmental material.

The weakness of *ACT2 *in soybean, rice, potato and sugarcane has been noted previously [32,37,39,40], while *ACT2/7 *was seen to be rather variable in *A. thaliana *[9]. However, *ACT2/7 *was judged to be the most stable of a set of ten conventional housekeeping genes across 21 soybean samples, covering a range of developmental stages [36]. Similarly, *TUB *performed poorly as a reference gene in grape, potato and soybean [16,36,39]. *UBQ10*, which ranked poorly in the present experiments, was previously deemed unsatisfactory as a reference in soybean [36] and in grape [16], but enjoyed very stable expression in *A. thaliana *and *Brachypodium sp*. [9,33]. *EF1b *was among the most stable genes both in this study and in a previous study of soybean [36], while in both potato and rice, *EF1α *was very stably expressed under conditions of biotic and abiotic stress [39]. The same gene was also identified as being highly stable in its expression across tissues of rice [31], but was unstable across tissues and organs of tomato at various developmental stages [38]. *TUA5 *was identified as being highly stable across development in soybean [36], while in poplar, *TUA *was very stably expressed across different tissues [41]. Here, *TUA5 *expression was hardly affected by changes in photoperiod. Globally, the best-performing genes were *SKIP16, UKN1, UKN2 *and *TIP41*, while the worst were *PEPKR1 *and *HDC*. *TIP41 *and *UKN2 *have been noted as showing stable expression across tissues and development in both tomato [38] and aspen [15]. However, *TIP41 *performed poorly during grape berry development [16], and in the roots and leaves of *A. thaliana *plants suffering cadmium or copper stress [30]. In aspen cambial cells, *UKN2 *expression was too unstable for the gene to be used for normalization [15]. Thus, overall, while certain reference genes are stably expressed in one plant species, they may not be well suited for use in others. As a consequence, prior validation of reference genes needs to be carried out under the specific experimental conditions to be applied in gene expression studies.

We report the application of various mathematical and statistical models to minimize bias in the quantification of gene expression in soybean. The first was a conventional statistical test to calculate the coefficient of variance (CV) of Cq values, which allowed an assessment of an individual gene's expression stability. But, due to its low sensitivity and reliability, this method can not clearly define the most stably expressed reference genes. The second exploited geNorm software [17], which showed that the stability of the various candidate reference genes varied considerably across the sets of samples (Figure 1). The third used the alternative program, NormFinder, which ranks the reference genes according to their expression stability [21]. The ranking of genes as revealed by NormFinder was mostly identical to that generated by geNorm (Table 3). Except for *TUB4*, all the candidate reference genes were represented in the Genevestigator database [55], and most of the expression patterns revealed by Genevestigator microarray data were consistent with the outputs of geNorm and NormFinder in the present data set (Additional file 6 and 7).

It has been argued that co-regulation of genes may confound geNorm analyses, because of the software's tendency to select the genes with a similar expression profile [21]. Among the set of genes tested, two pairs (*TUA5/TUB4 *and *ACT2/7/ACT11*) belong to a particular gene family, and thus may be prone to co-regulation. But the possibility that *ACT *and *TUA *may be co-regulated is unlikely in this study (Figure 3), given that *ACT11 *and *TUA5 *were consistently ranked above *ACT2/7 *and *TUB4 *except that *TUB4 *ranked above *TUA5 *in different cultivars.

The transcript abundance of many genes is, like *GmFTL3*, never very high, so any variation in their expression pattern is inevitably subtle. In this study, we normalized the expression of *GmFTL3 *with a total of seven normalization factors using individual or combinations of two, three and four control genes, and got similar patterns even though the levels of the abundance were different. But normalization with the combination of more genes resulted in improved accuracy. It suggests that the number of reference genes needed to be employed is dependent on the considerations of a researcher's purpose. That is, if one just wants to show a rough expression mode of genes, one reference gene may be enough if this reference gene was confirmed as a stable expressed gene. However, if the researcher hopes to compare the expression among different samples or to accurate the expression level, more reference genes (dependent on the geNorm threshold of 0.15) must be taken. This may be partially explained by that the geNorm threshold is not a strict cut-off and that the observed trend of changing pairwise variation values is equally informative [17,33,56].

## Conclusion

In the present study, we have investigated the expression of 14 candidate reference genes across a large number of soybean samples in an attempt to identify those most suitable for normalizing gene expression. No gene was consistently superior to the others, but most novel genes were better than the conventionally used housekeeping genes in terms of their expression stability. A combination of the three genes SKIP16, UKN1 and UKN2 provided the most robust platform for transcript normalization across experimental conditions in this study.

## Methods

### Plant Materials

The soybean cultivar Kennong18 (KN18) was used for most experiments. Plants were grown in a growth chamber under short day conditions (8 h light/16 h dark) at a temperature 25°C - 28°C. Seedling tissues were harvested before the expansion of the unifoliolate leaf. The root, hypocotyl, epicotyl, cotyledon, unifoliolate leaf and shoot apex (including the apical meristem and immature leaves) were sampled when the unifoliolate leaves had become fully expanded (about two weeks after sowing). A further sample of the root, along with the stem, unifoliolate leaves, various trifoliolate and lateral leaves, the petiole and the flowers were harvested when the fourth trifoliolate had become fully expanded (45 days after sowing, flowering onset). Pods and seeds were sampled at seven, 14 and 21 days after flowering, and at maturity. The aerial part of plants was also harvested respectively when the unifoliolate, first, second, third trifoliolate, and fourth trifoliolate were fully expanded (Additional file 5, indicated in yellow and green). To study the effect of altering the photoperiod, seedlings were exposed to either a long day (LD, 18 h light/6 h dark) or a short day (SD, 8 h light/16 h dark) regime. Fully expanded unifoliolate leaves were collected at 4 h intervals over 48 h, then the seedlings were transferred to either constant white light (LD) or constant darkness (SD), and the unifoliolate leaves re-sampled at 4 h intervals over a further 48 h (Additional file 5, indicated in grey). The effect of exposure to either red (RL) or blue (BL) light was monitored in etiolated seedlings subjected to red (Red-LED, 658 nm) or blue (Blue-LED, 436 nm) light in a growth chamber under LD conditions. The unifoliolate leaves were harvested at 4 h intervals over 48 h (Additional file 5, indicated in red and blue). Six further soybean cultivars were included: Heihe 27 (HH27), Zhonghuang 13 (ZH13), Jidou 12 (JD12), Tiefeng 31(TF31), Suinong 14 (SN14) and Fudou 1 (FD1). These seedlings were grown under either SD or LD conditions and the unifoliolate leaves were sampled 30 min before the lights were turned off (Additional file 5, indicated in purple). Totally, the experimental samples comprised 44 at various stages of development, 60 exposed to various photoperiod treatments, and 12 involving six different cultivars (Additional file 5). All samples were immediately frozen in liquid nitrogen and stored at -80°C until required.

### Total RNA isolation and cDNA synthesis

Total RNA was extracted using the TRIzol reagent (Invitrogen, CA, USA) according to the manufacturer's instructions. Alternatively, total RNA from the petioles was isolated by the CTAB method [57]. Only RNA preparations having an A260/A280 ratio of 1.8-2.0 and an A260/A230 ratio >2.0 were used for subsequent analysis. RNA integrity was verified by 2% agarose gel electrophoresis followed by SYBR Green staining. Before cDNA synthesis, the RNA was treated with RQ1 RNase-free DNase (Promega, Madison, WI, USA), according to the manufacturer's instructions, and first-strand cDNA synthesis was carried out using 4 μg RNA with the help of the RevertAid first strand cDNA synthesis kit (Fermentas, St. Leon-Roth, Germany) and oligo-dT primers, according to the manufacturer's protocol.

### Selection of candidate soybean genes

A set of 14 candidate reference genes was selected. This comprised seven conventionally used housekeeping genes; the soybean orthologs of the *A. thaliana *reference genes *TIP41 *(*At4G34270*), *HDC *(*At1G58050*) and *UKN2 *(*At4G33380*); and *SKIP16 *(*At1G06110*), *MTP *(*At2G41790*), *PEPKR1 *(*At1G12580*) and *UKN1 *(*At3G13410*), which were identified as potential reference genes via a soybean microarray gene expression analysis [37].

### PCR primer design and test of amplification efficiency

Primers were designed using Beacon Designer v7.0 (Premier Biosoft International, Palo Alto, California, USA) with melting temperatures 58-60°C, primer lengths 20-24 bp and amplicon lengths 60-134 bp. Experimental details are given in Table 2. Exon/intron boundaries were determined by aligning each cDNA sequence with its corresponding genomic sequence, downloaded from Phytozome . Five primer pairs were directed to locate on different exons or directly spanning exon-exon junction of each cDNA (Table 2). For each primer pair, reaction efficiency estimates were derived from a standard curve generated from a serial dilution of pooled cDNA. Mean quantification cycle (Cq) values of each ten-fold dilution were plotted against the logarithm of the cDNA dilution factor. An estimate of PCR efficiency was derived from the expression [10^(1/-S)^-1] × 100%, where S represents the slope of the linear regression [58].

### Real-time quantitative RT-PCR

RT-qPCR was conducted using an ABI StepOne Detection System (Applied Biosystems, USA), based on SYBR Premix Ex Taq polymerase (TaKaRa, Toyoto, Japan). Each 15 μl reaction comprised 4 μl template, 7.5 μl 2× SYBR Premix, 0.3 μl (200 nM) of each primer and 0.3 μl ROX. The reactions were subjected to an initial denaturation step of 95°C/10s, followed by 40 cycles of 95°C/5s and 60°C/60s. A melting curve analysis was performed at the end of the PCR run over the range 60-95°C, increasing the temperature stepwise by 0.5°C every 10s. Baseline and quantification cycle (Cq) were automatically determined using the StepOne Software v2.0. Zero template controls were included for each primer pair, and each PCR reaction was carried out in triplicate.

### Statistical analysis

Cq values were converted into relative quantities via the delta-Cq method using the sample with the lowest Cq as calibrator and incorporating the calculated amplification efficiencies for each primer pair (Table 2). The stability of reference gene expression was analysed with the geNorm (v3.5) and NormFinder (v0.953) software packages [19,20]. The former derives a stability measure (M), and via a stepwise exclusion of the least stable gene, creates a stability ranking. It also estimates the number of genes required to calculate a robust normalization factor (NF). NormFinder uses an ANOVA-based model to estimate intra- and inter-group variation, and combines these estimates to provide a direct measure of the variation in expression for each gene. All other statistical analyses were performed with SPSS (v13, SPSS Inc., Chicago, IL).

### Microarray data analysis

The stability of the reference gene set was validated using the 3,092 Genevestigator soybean genome microarray dataset, available at [55]. The Meta-Profile Analysis tool was used to represent each reference gene's expression stability according to its UniGene IDs (see Table 1).

## Abbreviations

RT-qPCR: quantitative real-time reverse transcriptase PCR; Cq: quantification cycle; *GAPDH*: glyceraldehyde-3-phosphate dehydrogenase; *ACT*: actin; *TUB*: β-tubulin; *TUA*: α-tubulin; *CYP*: cyclophilin; *EF1b*: eukaryotic translation elongation factor-1 β; *UBQ10*: ubiquitin 10; *SKIP16*: SKP1/ASK-interacting protein 16; *MTP*: metalloprotease; *PEPKR1*: phosphoenolpyruvate carboxylase-related kinase 1; *HDC*: helicase domain containing; *TIP41*: TIP41-like gene; *UKN1*, *UKN2*:genes of unknown function; CV: coefficient of variation; ANOVA: analysis of variance; NF: normalization factor.

## Authors' contributions

RH performed all the experimental procedures, data analysis and drafted the manuscript. CF participated in the statistical analysis and helped to draft the manuscript. HL and QZ provide the samples and participated in RNA and cDNA preparation. YF designed the project, supervised the study and critically revised the manuscript. All authors read and approved the final manuscript.

## Supplementary Material

Additional file 1 **List of reference genes used for gene expression studies in soybean**. The list comprises 54 hits from a search (January 2001 to March 2009) of PubMed, using "soybean" and "gene expression" as keywords. Click here for file

Additional file 2 **The transcription profiles of individual reference genes given as absolute Cq values across all samples**. The scatter plots show the expression levels of the various reference genes. Values are given in the form of quantification cycle numbers (Cq values). Click here for file

Additional file 3 **Representative amplification plots and melting curves obtained in the RT-qPCR efficiency test**. Four to five ten-fold serial dilutions were plotted against the logarithm of cDNA template concentration. Amplification plots and melting curve images were collected using StepOne software v2.0 (Applied Biosystems). Click here for file

Additional file 4 **RT-qPCR primer efficiency plots**. Mean quantification cycle (Cq) values of each set of ten-fold serial dilution plotted against the logarithm of cDNA template concentration. The reaction efficiency (E) is given by [10^(1/-S)^-1] × 100%, where S represents the slope of the linear regression line. Click here for file

Additional file 5 **Tissue/organ sample sets used for the analysis of gene expression**. See Methods section for details. Click here for file

Additional file 6 **Expression profiling of six conventional housekeeping genes, using microarray data derived from Genevestigator**. The Meta-Profile Analysis tool was used to produce expression profiling from representative UniGene IDs. No probes available for TUB4. Click here for file

Additional file 7 **Expression profiling of seven new reference genes tested from Genevestigator microarray data**. The Meta-Profile Analysis tool was used to produce expression profiling from representative UniGene IDs. Click here for file
