# Supplementary material for: Evaluation of putative reference genes for gene expression normalization in soybean by quantitative real-time RT-PCR
Source: BMC Mol Biol. 2009 Sep 28;10:93. doi: 10.1186/1471-2199-10-93 (PMC2761916; doi:10.1186/1471-2199-10-93)
Supplement: Additional file 1 — List of reference genes used for gene expression studies in soybean. The list comprises 54 hits from a search (January 2001 to March 2009) of PubMed, using "soybean" and "gene expression" as keywords. [file 1471-2199-10-93-S1.PDF]

Additional file 1: List of reference genes used for gene expression studies in soybean.

No.	cDNA types	Reference genes	Q/Semi-Q	References
1	Root, leaf, shoot apices and dry seeds	18S rRNA	Q	Eller et al (2006)
2	Juvenile leaves at different developmental stages, different tissues, and leaves treated with 6-BA	18S rRNA	Semi-Q	Li et al (2006)
3	Root and shoot tissues treated with ACC, ET and JA	18S rRNA	Q	Mazarei et al (2007)
4	Hypocotyls, roots, leaves, flowers and nodules	18S rRNA	Semi-Q	Phartiyal et al (2008)
5	Nodules at different developmental stages, nodules under illumination and dark treatment	18S rRNA	Q	Xu et al (2007)
6	Different tissues, different nitrogen concentrations treatments	18S rRNA	Semi-Q	Yokoyama et al (2001)
7	Different tissues infected with *Bradyrhizobium japonicum*	25S rRNA	Q	Nontachaiyapoom et al (2007)
8	Leaves infected with *Bradyrhizobium japonicum*	Actin	Q	Nontachaiyapoom et al (2007)
9	Seeds, leaves, roots and pods	Actin	Semi-Q	Cascardo et al (2001)
10	Leaves exposed to elevated CO_2 and O_3, and infested with Japanese beetles	Actin	Q	Casteel et al (2008)
11	Cell cultures treated with Actinomicin-D and Dark / illumination	Actin	Semi-Q	Collados et al (2006)
12	Leaves of 4-week-old plants	Actin	Semi-Q	Gao et al (2007)
13	Shoot apical meristem (SAM)	Actin	Semi-Q	Haerizadeh et al (2009)
14	Developmental stages, tissues	Actin	Semi-Q	Huang et al (2006)
15	Cotyledons treated with tunicamycin (TM) and AZC	Actin	Q	Kamauchi et al (2008)
16	Leaves and root from seedlings inoculated with *Bradyrhizobium sp*., mutant	Actin	Q	Kinkema et al (2008)
17	Developing seeds and other tissues	Actin	Semi-Q	Li et al (2008)
18	Developmental stages, tissues	Actin	Semi-Q	Li et al (2007)
19	Developmental steges, tissues	Actin	Semi-Q	Meng et al (2007)
20	Tissues and developmental stages	Actin	Semi-Q	Terauchi et al (2004)
21	Leaves, stem and root	Actin 3	Semi-Q	Kongrit et al (2007)
22	Tissues, circadian, stress (ABA, epiBL)	Actin 4	Q	Zhao et al (2008)
23	Developing seeds	EF1a	Semi-Q	Nunes et al (2006)
24	Leaves, stems, flowers, different embryos and somatic embryo culture	EF1a	Semi-Q	Thakare et al (2008)
25	Root infected with soybean cyst nematode (SCN)	EF1ß	Q	Tucker et al (2007)
26	Leaves, shoot apices, floral apices and root	IOTA	Semi-Q	Yamamoto et al (2001)
27	Leaves treated with auxin, IAA, 2,4-D etc	PEPC	Q	Kelley et al (2004)
28	Leaves infected with soybean mosaic virus (SMV)	Tublin beta	Semi-Q	Wang et al (2005)
29	Tissues, leaves treated with ABA, cold (4?) , drought and salt	Tubulin	Semi-Q	Chen et al (2007)
30	Leaves, root and embryonic axes of seeds exposed to cold(4?)	Tubulin	Semi-Q	Cheng et al (2009)
31	Cell suspension cultures treated with SA and JA	Tubulin	Q	Eichhorn et al (2006)
32	Leaves, flowers, pods , aerial parts and underground parts	Tubulin	Semi-Q	Graham et al (2002)
33	Leaves treated with SA, soybean mosaic virus (SMV) and wounding	Tubulin	Semi-Q	He et al (2003)
34	Tissues, leaves ireated with NaCl, PEG, ABA and cold (4℃)	Tubulin	Semi-Q	Li et al (2005)

35	Leaves treated with ABA, cold, drought and salt treatments, different tissues	Tubulin	Semi-Q	Liao et al (2008)
36	Tissues, leaves treated with ABA, cold, drought and salt	Tubulin	Semi-Q	Liao et al (2008)
37	Tissues, leaves treated with ABA, cold, drought and salt	Tubulin	Semi-Q	Liao et al (2008)
38	Tissue, leaves treated with IAA, ABA, JA, GA and cytokinin	Tubulin	Semi-Q	Liu et al (2008)
39	Tissues, leaves under $Cacl_2$, NaCl, KCl, LiCl, ABA, PEG and Cold stress	Tubulin	Semi-Q	Luo et al (2005)
40	Leaves inoculated with P.*pachyrhizi*	Tubulin	Q	Panthee et al (2009)
41	Leaves inoculated with P.*pachyrhizi*	Tubulin	Q	Panthee et al (2007)
42	Cotyledons, roots, leaves, flowers, pods	Tubulin	Semi-Q	Schlueter et al (2006)
43	Leaves treated with SA and wounding	Tubulin	Semi-Q	Tian et al (2004)
44	Leaves, flowers and pods	Tubulin	Semi-Q	Wang et al (2007)
45	Leaves and seedlings under different photoperiod treatment (SD/LD, Red light, Blue light)	Tubulin	Q	Zhang et al (2008)
46	Seedlings treated with NaCl, drought, cold, SMV, ET, SA, JA, and ABA	Tubulin	Semi-Q	Zhang et al (2008)
47	Leaves treated with NaCl, drought, and cold	Tubulin	Semi-Q	Zhou et al (2008)
48	Seedlings in phosphorus starvation and IAA treatment.	Tubulin alpha	Q	Shen et al (2006)
49	Tissues at different developmental stages	Tubulin beta	Semi-Q	Wang et al (2006)
50	Hypocotyls and roots inoculated with *Phytophthora sojae*, different cultivars	Tubulin 2	Semi-Q	Valer et al (2006)
51	Seedlings treated with wounding and hypocotyls infected with *Sclerotinia sclerotiorum*	UBQ	Semi-Q	D'Ovidio et al (2006)
52	Tissues in different photoperiod treatment	UBQ	Semi-Q	Delis et al (2006)
53	Pods and seeds at six developmental stages	UBQ	Semi-Q	Kim et al (2009)
54	Leaves at different photoperiodic treatment	UBQ	Semi-Q	Liu et al (2009)
55	Nodules under illumination, dark, shoot-detopping and stem girding treatment	UBQ	Semi-Q	Xu et al (2003)

Note: Q: Real-time quantitative RT-PCR; Semi-Q: Semi-quantitative RT-PCR; PEPC: Phosphoenolpyruvate carboxylases
ACC: 1-aminocyclopropane-1-carboxylic acid, JA: Jasmonic acid, SA: Salicylic acid, ET: Ethylene, AZC: L-azetidine-2-carboxylic acid, ABA: Abscisic acid, IAA: Indole-3-acetic acid

References:

1. Eller MH, Warner AL, Knap HT: **Genomic organization and expression analyses of putrescine pathway genes in soybean**. *Plant Physiol Biochem* 2006, **44**(1):49-57.
2. Li XP, Gan R, Li PL, Ma YY, Zhang LW, Zhang R, Wang Y, Wang NN: **Identification and functional characterization of a leucine-rich repeat receptor-like kinase gene that is involved in regulation of soybean leaf senescence**. *Plant Mol Biol* 2006, **61**(6):829-844.
3. Mazarei M, Elling AA, Maier TR, Puthoff DP, Baum TJ: **GmEREBP1 is a transcription factor activating defense genes in soybean and Arabidopsis**. *Mol Plant Microbe Interact* 2007, **20**(2):107-119.
4. Phartiyal P, Kim WS, Cahoon RE, Jez JM, Krishnan HB: **The role of 5'-adenylylsulfate reductase in the sulfur assimilation pathway of soybean: molecular**

cloning, kinetic characterization, and gene expression. *Phytochemistry* 2008, **69**(2):356-364.

5. Xu W, Sato SJ, Clemente TE, Chollet R: **The PEP-carboxylase kinase gene family in Glycine max (GmPpcK1-4): an in-depth molecular analysis with nodulated, non-transgenic and transgenic plants**. *Plant J* 2007, **49**(5):910-923.
6. Yokoyama T, Kodama N, Aoshima H, Izu H, Matsushita K, Yamada M: **Cloning of a cDNA for a constitutive NRT1 transporter from soybean and comparison of gene expression of soybean NRT1 transporters**. *Biochim Biophys Acta* 2001, **1518**(1-2):79-86.
7. Nontachaiyapoom S, Scott PT, Men AE, Kinkema M, Schenk PM, Gresshoff PM: **Promoters of orthologous Glycine max and Lotus japonicus nodulation autoregulation genes interchangeably drive phloem-specific expression in transgenic plants**. *Mol Plant Microbe Interact* 2007, **20**(7):769-780.
8. Cascardo JC, Buzeli RA, Almeida RS, Otoni WC, Fontes EP: **Differential expression of the soybean BiP gene family**. *Plant Sci* 2001, **160**(2):273-281.
9. Casteel CL, O'Neill BF, Zavala JA, Bilgin DD, Berenbaum MR, Delucia EH: **Transcriptional profiling reveals elevated CO2 and elevated O3 alter resistance of soybean (Glycine max) to Japanese beetles (Popillia japonica)**. *Plant Cell Environ* 2008, **31**(4):419-434.
10. Collados R, Andreu V, Picorel R, Alfonso M: **A light-sensitive mechanism differently regulates transcription and transcript stability of omega3 fatty-acid desaturases (FAD3, FAD7 and FAD8) in soybean photosynthetic cell suspensions**. *FEBS Lett* 2006, **580**(20):4934-4940.
11. Gao XR, Wang GK, Su Q, Wang Y, An LJ: **Phytase expression in transgenic soybeans: stable transformation with a vector-less construct**. *Biotechnol Lett* 2007, **29**(11):1781-1787.
12. Haerizadeh F, Wong CE, Singh MB, Bhalla PL: **Genome-wide analysis of gene expression in soybean shoot apical meristem**. *Plant Mol Biol* 2009, **69**(6):711-727.
13. Huang F, Chi Y, Meng Q, Gai J, Yu D: **GmZFP1 encoding a single zinc finger protein is expressed with enhancement in reproductive organs and late seed development in soybean (Glycine max)**. *Mol Biol Rep* 2006, **33**(4):279-285.
14. Kamauchi S, Wadahama H, Iwasaki K, Nakamoto Y, Nishizawa K, Ishimoto M, Kawada T, Urade R: **Molecular cloning and characterization of two soybean protein disulfide isomerases as molecular chaperones for seed storage proteins**. *FEBS J* 2008, **275**(10):2644-2658.
15. Kinkema M, Gresshoff PM: **Investigation of downstream signals of the soybean autoregulation of nodulation receptor kinase GmNARK**. *Mol Plant Microbe Interact* 2008, **21**(10):1337-1348.
16. Li L, Wang X, Gai J, Yu D: **Isolation and characterization of a seed-specific isoform of microsomal omega-6 fatty acid desaturase gene (FAD2-1B) from soybean**. *DNA Seq* 2008, **19**(1):28-36.
17. Li L, Wang X, Gai J, Yu D: **Molecular cloning and characterization of a novel microsomal oleate desaturase gene from soybean**. *J Plant Physiol* 2007, **164**(11):1516-1526.
18. Meng Q, Zhang C, Gai J, Yu D: **Molecular cloning, sequence characterization and tissue-specific expression of six NAC-like genes in soybean (Glycine max**

(L.) Merr.). *J Plant Physiol* 2007, **164**(8):1002-1012.

19. Terauchi K, Asakura T, Nishizawa NK, Matsumoto I, Abe K: **Characterization of the genes for two soybean aspartic proteinases and analysis of their different tissue-dependent expression**. *Planta* 2004, **218**(6):947-957.
20. Kongrit D, Jisaka M, Iwanaga C, Yokomichi H, Katsube T, Nishimura K, Nagaya T, Yokota K: **Molecular cloning and functional expression of soybean allene oxide synthases**. *Biosci Biotechnol Biochem* 2007, **71**(2):491-498.
21. Zhao L, Luo Q, Yang C, Han Y, Li W: **A RAV-like transcription factor controls photosynthesis and senescence in soybean**. *Planta* 2008, **227**(6):1389-1399.
22. Nunes AC, Vianna GR, Cuneo F, Amaya-Farfan J, de Capdeville G, Rech EL, Aragao FJ: **RNAi-mediated silencing of the myo-inositol-1-phosphate synthase gene (GmMIPS1) in transgenic soybean inhibited seed development and reduced phytate content**. *Planta* 2006, **224**(1):125-132.
23. Thakare D, Tang W, Hill K, Perry SE: **The MADS-domain transcriptional regulator AGAMOUS-LIKE15 promotes somatic embryo development in Arabidopsis and soybean**. *Plant Physiol* 2008, **146**(4):1663-1672.
24. Tucker ML, Burke A, Murphy CA, Thai VK, Ehrenfried ML: **Gene expression profiles for cell wall-modifying proteins associated with soybean cyst nematode infection, petiole abscission, root tips, flowers, apical buds, and leaves**. *J Exp Bot* 2007, **58**(12):3395-3406.
25. Yamamoto E, Knap HT: **Soybean receptor-like protein kinase genes: paralogous divergence of a gene family**. *Mol Biol Evol* 2001, **18**(8):1522-1531.
26. Kelley KB, Lambert KN, Hager AG, Riechers DE: **Quantitative expression analysis of GH3, a gene induced by plant growth regulator herbicides in soybean**. *J Agric Food Chem* 2004, **52**(3):474-478.
27. Wang YJ, Li YD, Luo GZ, Tian AG, Wang HW, Zhang JS, Chen SY: **Cloning and characterization of an HDZip I gene GmHZ1 from soybean**. *Planta* 2005, **221**(6):831-843.
28. Chen M, Wang QY, Cheng XG, Xu ZS, Li LC, Ye XG, Xia LQ, Ma YZ: **GmDREB2, a soybean DRE-binding transcription factor, conferred drought and high-salt tolerance in transgenic plants**. *Biochem and Biophy Res Comm* 2007, **353**(2):299-305.
29. Cheng L, Huan S, Sheng Y, Hua X, Shu Q, Song S, Jing X: **GMCHI, cloned from soybean [Glycine max (L.) Meer.], enhances survival in transgenic Arabidopsis under abiotic stress**. *Plant Cell Rep* 2009, **28**(1):145-153.
30. Eichhorn H, Klinghammer M, Becht P, Tenhaken R: **Isolation of a novel ABC-transporter gene from soybean induced by salicylic acid**. *J Exp Bot* 2006, **57**(10):2193-2201.
31. Graham MA, Marek LF, Shoemaker RC: **Organization, expression and evolution of a disease resistance gene cluster in soybean**. *Genetics* 2002, **162**(4):1961-1977.
32. He CY, Tian AG, Zhang JS, Zhang ZY, Gai JY, Chen SY: **Isolation and characterization of a full-length resistance gene homolog from soybean**. *Theor Appl Genet*

2003, **106**(5):786-793.

33. Li XP, Tian AG, Luo GZ, Gong ZZ, Zhang JS, Chen SY: **Soybean DRE-binding transcription factors that are responsive to abiotic stresses**. *Theor Appl Genet* 2005, **110**(8):1355-1362.

34. Liao Y, Zou HF, Wei W, Hao YJ, Tian AG, Huang J, Liu YF, Zhang JS, Chen SY: **Soybean GmbZIP44, GmbZIP62 and GmbZIP78 genes function as negative regulator of ABA signaling and confer salt and freezing tolerance in transgenic Arabidopsis**. *Planta* 2008, **228**(2):225-240.

35. Liao Y, Zou HF, Wang HW, Zhang WK, Ma B, Zhang JS, Chen SY: **Soybean GmMYB76, GmMYB92, and GmMYB177 genes confer stress tolerance in transgenic Arabidopsis plants**. *Cell Res* 2008, **18**(10):1047-1060.

36. Liao Y, Zhang JS, Chen SY, Zhang WK: **Role of soybean GmbZIP132 under abscisic acid and salt stresses**. *J Integr Plant Biol* 2008, **50**(2):221-230.

37. Liu J, Ha D, Xie Z, Wang C, Wang H, Zhang W, Zhang J, Chen S: **Ectopic expression of soybean GmKNT1 in Arabidopsis results in altered leaf morphology and flower identity**. *J Genet Genomics* 2008, **35**(7):441-449.

38. Luo GZ, Wang HW, Huang J, Tian AG, Wang YJ, Zhang JS, Chen SY: **A putative plasma membrane cation/proton antiporter from soybean confers salt tolerance in Arabidopsis**. *Plant Mol Biol* 2005, **59**(5):809-820.

39. Panthee DR, Marois JJ, Wright DL, Narvaez D, Yuan JS, Stewart CN, Jr.: **Differential expression of genes in soybean in response to the causal agent of Asian soybean rust (Phakopsora pachyrhizi Sydow) is soybean growth stage-specific**. *Theor Appl Genet* 2009, **118**(2):359-370.

40. Panthee DR, Yuan JS, Wright DL, Marois JJ, Mailhot D, Stewart CN, Jr.: **Gene expression analysis in soybean in response to the causal agent of Asian soybean rust (Phakopsora pachyrhizi Sydow) in an early growth stage**. *Funct Integr Genomics* 2007, **7**(4):291-301.

41. Schlueter JA, Scheffler BE, Schlueter SD, Shoemaker RC: **Sequence conservation of homeologous bacterial artificial chromosomes and transcription of homeologous genes in soybean (Glycine max L. Merr.)**. *Genetics* 2006, **174**(2):1017-1028.

42. Tian AG, Luo GZ, Wang YJ, Zhang JS, Gai JY, Chen SY: **Isolation and characterization of a Pti1 homologue from soybean**. *J Exp Bot* 2004, **55**(396):535-537.

43. Wang HW, Zhang B, Hao YJ, Huang J, Tian AG, Liao Y, Zhang JS, Chen SY: **The soybean Dof-type transcription factor genes, GmDof4 and GmDof11, enhance lipid content in the seeds of transgenic Arabidopsis plants**. *Plant J* 2007, **52**(4):716-729.

44. Zhang Q, Li H, Li R, Hu R, Fan C, Chen F, Wang Z, Liu X, Fu Y, Lin C: **Association of the circadian rhythmic expression of GmCRY1a with a latitudinal cline in photoperiodic flowering of soybean**. *Proc Natl Acad Sci U S A* 2008, **105**(52):21028-21033.

45. Zhang G, Chen M, Chen X, Xu Z, Guan S, Li LC, Li A, Guo J, Mao L, Ma Y: **Phylogeny, gene structures, and expression patterns of the ERF gene family in soybean (Glycine max L.)**. *J Exp Bot* 2008, **59**(15):4095-4107.

46. Zhou QY, Tian AG, Zou HF, Xie ZM, Lei G, Huang J, Wang CM, Wang HW, Zhang JS, Chen SY: **Soybean WRKY-type transcription factor genes, GmWRKY13,**

GmWRKY21, and GmWRKY54, confer differential tolerance to abiotic stresses in transgenic Arabidopsis plants. *Plant Biotechnol J* 2008, **6**(5):486-503.

47. Shen H, Chen J, Wang Z, Yang C, Sasaki T, Yamamoto Y, Matsumoto H, Yan X: **Root plasma membrane H+-ATPase is involved in the adaptation of soybean to phosphorus starvation**. *J Exp Bot* 2006, **57**(6):1353-1362.
48. Wang HW, Zhang JS, Gai JY, Chen SY: **Cloning and comparative analysis of the gene encoding diacylglycerol acyltransferase from wild type and cultivated soybean**. *Theor Appl Genet* 2006, **112**(6):1086-1097.
49. Valer K, Fliegmann J, Frohlich A, Tyler BM, Ebel J: **Spatial and temporal expression patterns of Avr1b-1 and defense-related genes in soybean plants upon infection with Phytophthora sojae**. *FEMS Microbiol Lett* 2006, **265**(1):60-68.
50. D'Ovidio R, Roberti S, Di Giovanni M, Capodicasa C, Melaragni M, Sella L, Tosi P, Favaron F: **The characterization of the soybean polygalacturonase-inhibiting proteins (Pgip) gene family reveals that a single member is responsible for the activity detected in soybean tissues**. *Planta* 2006, **224**(3):633-645.
51. Delis C, Dimou M, Flemetakis E, Aivalakis G, Katinakis P: **A root- and hypocotyl-specific gene coding for copper-containing amine oxidase is related to cell expansion in soybean seedlings**. *J Exp Bot* 2006, **57**(1):101-111.
52. Kim S, Kim SJ, Shin YJ, Kang JH, Kim MR, Nam KH, Lee MS, Lee SH, Kim YH, Hong SK *et al*: **An atypical soybean leucine-rich repeat receptor-like kinase, GmLRK1, may be involved in the regulation of cell elongation**. *Planta* 2009, **229**(4):811-821.
53. Liu H, Wang H, Gao P, Xu J, Xu T, Wang J, Wang B, Lin C, Fu YF: **Analysis of clock gene homologs using unifoliolates as target organs in soybean (Glycine max)**. *J Plant Physiol* 2009, **166**(3):278-289.
54. Xu W, Zhou Y, Chollet R: **Identification and expression of a soybean nodule-enhanced PEP-carboxylase kinase gene (NE-PpcK) that shows striking up-/down-regulation in vivo**. *Plant J* 2003, **34**(4):441-452.
