# Supplementary figures and images for: Evaluation of putative reference genes for gene expression normalization in soybean by quantitative real-time RT-PCR
Source: BMC Mol Biol. 2009 Sep 28;10:93. doi: 10.1186/1471-2199-10-93 (PMC2761916; doi:10.1186/1471-2199-10-93)

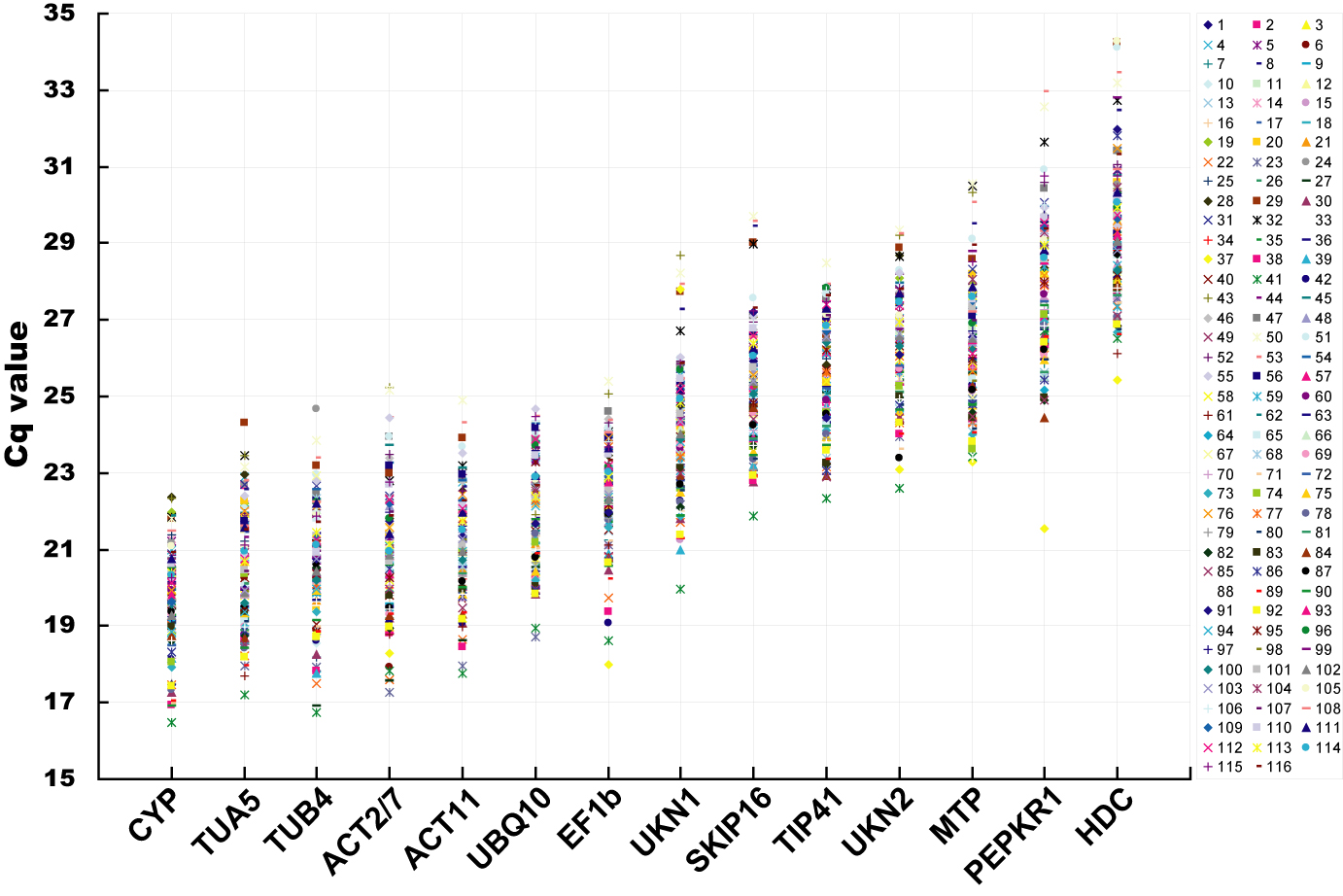

Supplement: Additional file 2 — The transcription profiles of individual reference genes given as absolute Cq values across all samples. The scatter plots show the expression levels of the various reference genes. Values are given in the form of quantification cycle numbers (Cq values). [file 1471-2199-10-93-S2.JPEG]

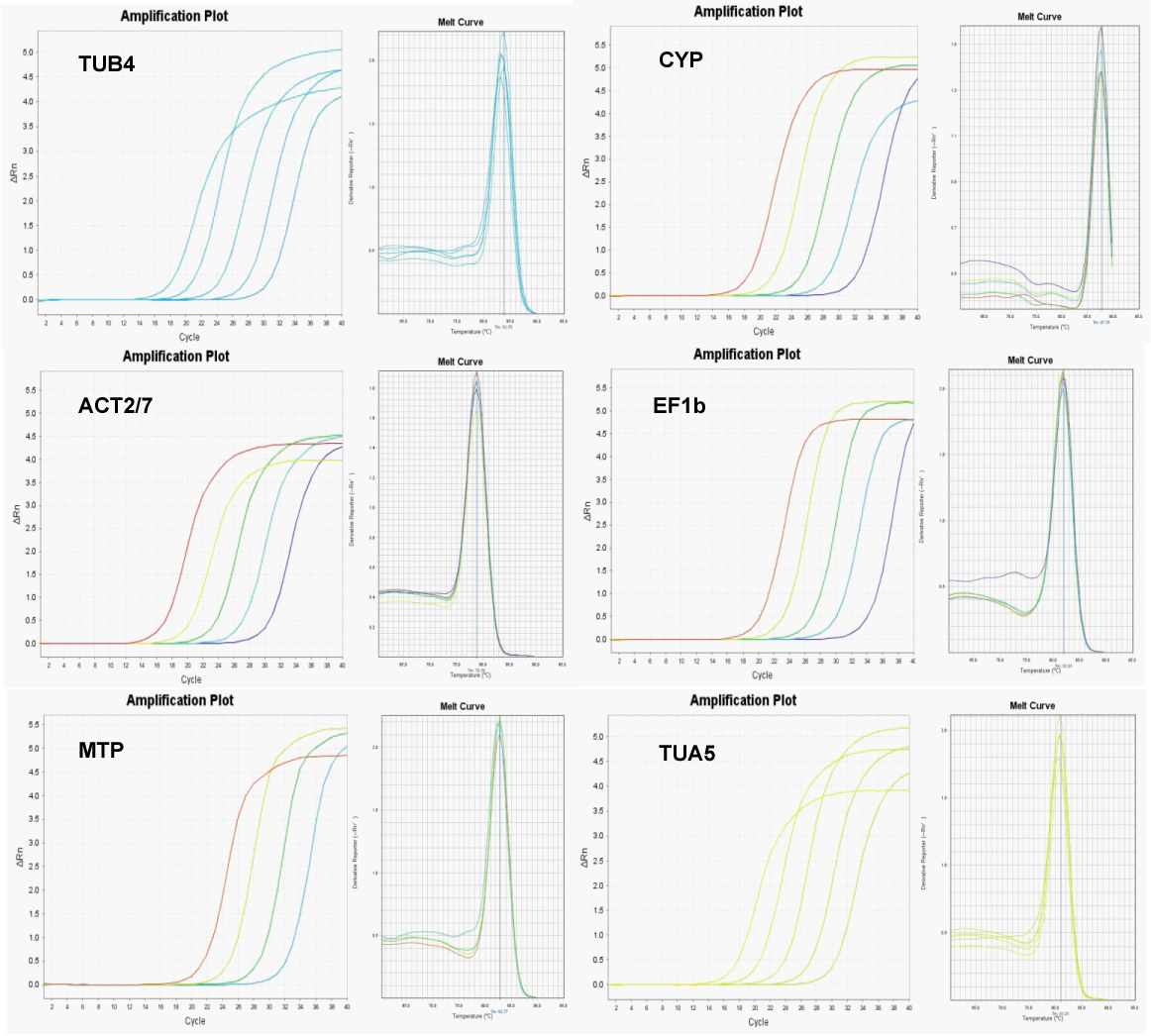

Supplement: Additional file 3 — Representative amplification plots and melting curves obtained in the RT-qPCR efficiency test. Four to five ten-fold serial dilutions were plotted against the logarithm of cDNA template concentration. Amplification plots and melting curve images were collected using StepOne software v2.0 (Applied Biosystems). [file 1471-2199-10-93-S3.JPEG]

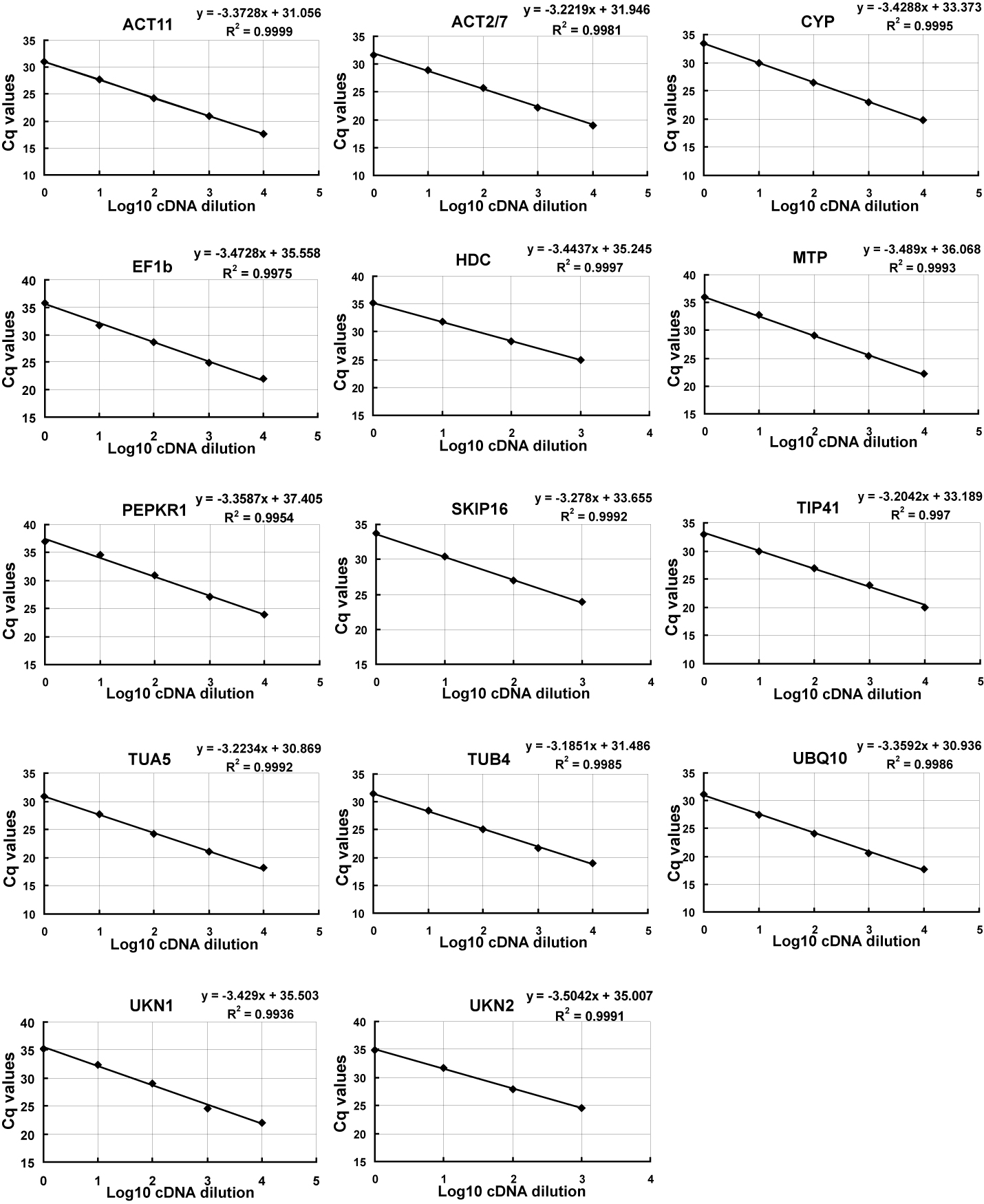

Supplement: Additional file 4 — RT-qPCR primer efficiency plots. Mean quantification cycle (Cq) values of each set of ten-fold serial dilution plotted against the logarithm of cDNA template concentration. The reaction efficiency (E) is given by [10(1/-S)-1] × 100%, where S represents the slope of the linear regression line. [file 1471-2199-10-93-S4.JPEG]

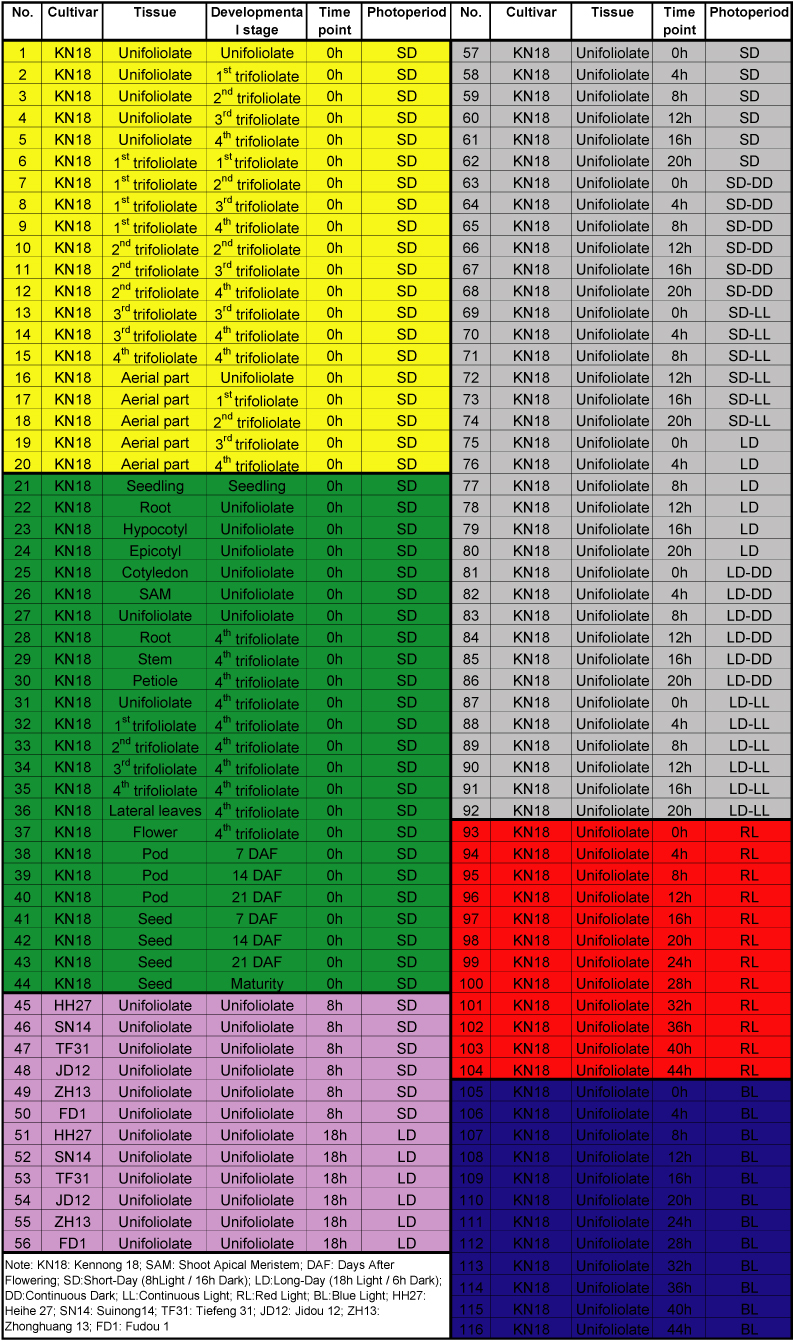

Supplement: Additional file 5 — Tissue/organ sample sets used for the analysis of gene expression. See Methods section for details. [file 1471-2199-10-93-S5.JPEG]
