# Supplementary material for: Evaluation of putative reference genes for gene expression normalization in soybean by quantitative real-time RT-PCR
Source: BMC Mol Biol. 2009 Sep 28;10:93. doi: 10.1186/1471-2199-10-93 (PMC2761916; doi:10.1186/1471-2199-10-93)

Additional file 7: Expression profiling of seven new reference genes tested from Genevestigator microarray data.

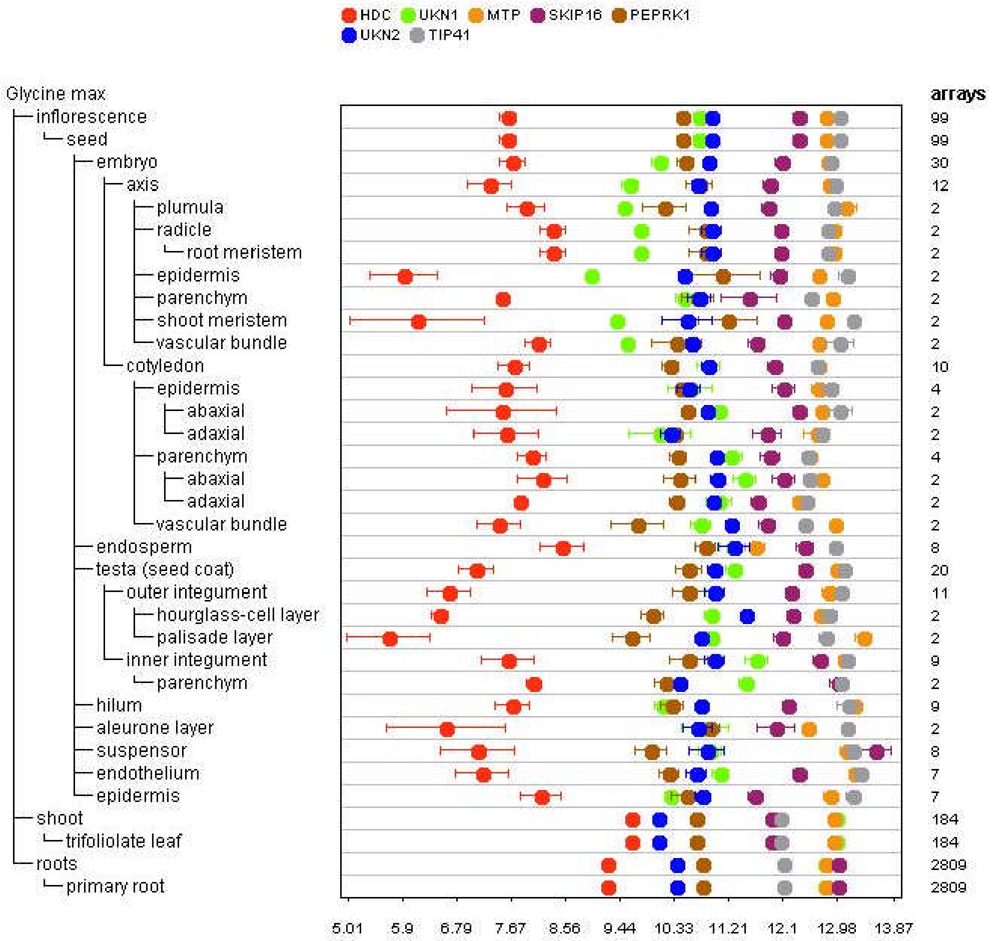

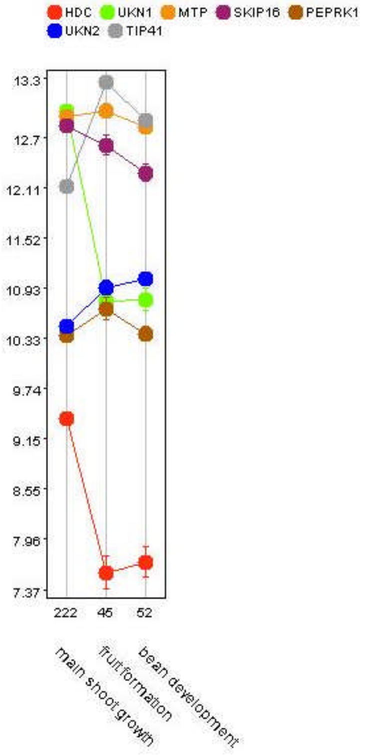

Supplement: Additional file 7 — Expression profiling of seven new reference genes tested from Genevestigator microarray data. The Meta-Profile Analysis tool was used to produce expression profiling from representative UniGene IDs. [file 1471-2199-10-93-S7.PDF]
